# A Protein Nanopore-Based Approach for Bacteria Sensing

**DOI:** 10.1186/s11671-016-1715-z

**Published:** 2016-11-15

**Authors:** Aurelia Apetrei, Andrei Ciuca, Jong-kook Lee, Chang Ho Seo, Yoonkyung Park, Tudor Luchian

**Affiliations:** 1 Department of Physics, Alexandru I. Cuza University, Iasi, Romania; 2 Research Center for Proteineous Materials, Chosun University, Gwangju, South Korea; 3 Department of Bioinformatics, Kongju National University, Kongju, South Korea

**Keywords:** Protein nanopore, α-Hemolysin, Gram-negative bacteria, Antimicrobial peptides, Bacteria biosensing

## Abstract

We present herein a first proof of concept demonstrating the potential of a protein nanopore-based technique for real-time detection of selected Gram-negative bacteria (*Pseudomonas aeruginosa* or *Escherichia coli*) at a concentration of 1.2 × 10^8^ cfu/mL. The anionic charge on the bacterial outer membrane promotes the electrophoretically driven migration of bacteria towards a single α-hemolysin nanopore isolated in a lipid bilayer, clamped at a negative electric potential, and followed by capture at the nanopore’s mouth, which we found to be described according to the classical Kramers’ theory. By using a specific antimicrobial peptide as a putative molecular biorecognition element for the bacteria used herein, we suggest that the detection system can combine the natural sensitivity of the nanopore-based sensing techniques with selective biological recognition, in aqueous samples, and highlight the feasibility of the nanopore-based platform to provide portable, sensitive analysis and monitoring of bacterial pathogens.

**Electronic supplementary material:**

The online version of this article (doi:10.1186/s11671-016-1715-z) contains supplementary material, which is available to authorized users.

## Background

One of the clearest and present dangers posed to health is the acquired microbial infection, which can spread widely, mainly due to the globalized food and beverage production and supply or in the form of biological warfare agents. While such outbreaks hit equally high-income countries as well as poor or developing ones, the latter category still remains more exposed to such risks, as the 2012 World Health Organization data from sub-Saharan Africa reported more than two million deaths attributed to a variety of infectious diseases [1].

This alone calls for the delivery of cheap, easy-to-use, and improved microbial detection methods. To detect and identify pathogens, among widely used approaches are the traditional sample cultivation protocols, enzyme-linked immunosorbent assay, immunomagnetic separation, surface plasmon resonance detection, and nucleic acid-based polymerase chain reaction technologies [2–5].

Despite their proven potential, the abovementioned techniques are plagued by a number of drawbacks that hinder them from becoming the method of choice when it comes to achieving portable and rapid pathogens detection, e.g., their implementation is time-consuming, costly, and labor-intensive, and it requires well-equipped, sterile laboratories and highly trained personnel [6, 7].

Thus, efforts were devoted to developing miniaturized diagnostic systems based on microfluidic platforms or nanomaterials for pathogen detection and analysis that circumvent the necessity of sophisticated infrastructure and sample preparation procedures, paving the way for a faster diagnosis with reduced implementation costs, while simultaneously providing reliable epidemiological data [8–14].

One of the newest paradigms applicable for sensitive, selective and high-throughput detection of biological-active compounds relies on protein or solid-stated nanopore-based biosensors, which have emerged as materials with powerful analytic capabilities for biosensing applications. In a nutshell, the idea behind the nanopore-based biosensors relies on the fact that the single macromolecule capture, and subsequent interactions of the molecules and the pore, produces a displacement of electrolytes in the channel, leading to a sudden decrease in conductivity which can be resolved and recorded using low noise feedback-loop operational amplifiers. Due to the fact that such characteristic changes in the nanopore-mediated current depend upon the physico-chemical and topological features of the analyte, the analysis of the stochastic electric blockade signature can be used to reveal the concentration, identity, and other microscopic features of the analyte (e.g., diffusion coefficient, volume, charge) [15–18].

As vivid examples suggesting their usefulness in the realm of molecular detection, nanopores were successfully employed for RNA and DNA detection and analysis [19–22], peptides [23–25], protein detection and analysis [26–28], and small analytes like metal ions, amino acids, neurotransmitters, or antibiotic molecules belonging to the β-lactam family [29–35]. It is thus accepted that biosensors based upon nanopores or ion channels may shape the modern paradigms of medical care and drug screening, environmental monitoring, or food safety [36–38].

With direct relevance for the detection of pathogens, leading to the possible development of robust and portable solid-state nanopore-based biosensors, in recent papers, there were reported the detection of single HIV-1 particles [39], the rigid rod-shaped tobacco mosaic virus (TMV) [40], and the capture and translocation of the stiff filamentous virus fd, which may have further applications as fd virus is a marker of sewage contamination in ground water [41].

We present herein a first proof of concept, to our knowledge, for the detection of two different Gram-negative bacteria (i.e., *Pseudomonas aeruginosa* and *Escherichia coli*) with a single α-hemolysin (α-HL) protein pore embedded in a reconstituted lipid bilayer. By exploiting the binding efficiency towards these bacterial strains of an antimicrobial peptide developed in our laboratories, we probed the possibility of differentiating between the two types of bacteria with the α-HL nanopore. With further development, we suggest that the approach might be applied to the fabrication of bacterial sensor nanoarrays, enabling real-time detection of multiple strains of bacterial cells simultaneously.

## Methods

### Bacterial Culture

*E. coli* (ATCC 25922) and *P. aeruginosa* (ATCC 15692) were obtained from the American Type Culture Collection (Manassas, VA, USA). Bacterial cells were cultured at 37 °C in appropriate culture medium (Luria Broth or 0.05% NaCl with nutrient broth). The cells were counted (1 × 10^8^ cfu/mL) using a UV spectrophotometer. Next, the cells were pelleted by centrifugation at 4000×*g* for 5 min and washed with phosphate-buffered saline (PBS), and moisture was eliminated using a lyophilizer for 5 min.

### Scanning Electron Microscopy (SEM)

**Fig. 1:**
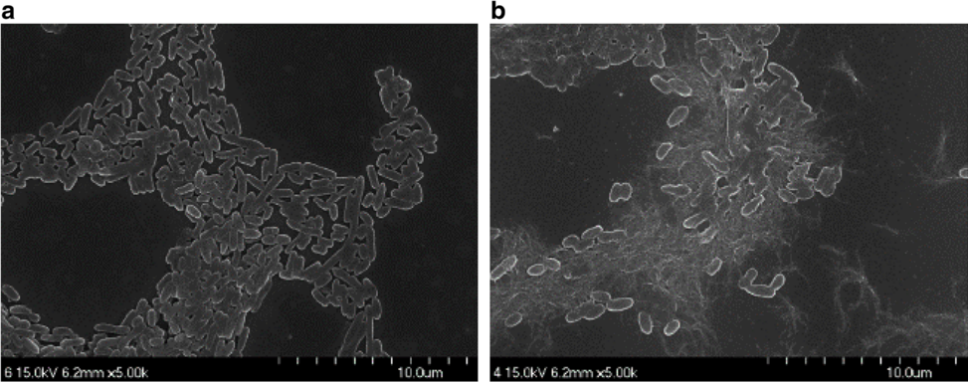
Morphological analysis of bacterial membrane using scanning electron microscopy (SEM). Representative pictures of **a** *E. coli* and **b** *P. aeruginosa* bacterial cells by SEM. *Scale bar* = 10 μm

Morphological features of bacterial cells were assessed by comparing *E. coli* and *P. aeruginosa* cells grown to mid-logarithmic growth phase at 37 °C and resuspended in 10 mM sodium phosphate buffer (pH 7.4). The cells were fixed with 4% paraformaldehyde for 2 h at 4 °C, dehydrated through a 50–100% ethanol series (10 min at each step), and coated with platinum. The cells were then examined under a scanning electron microscope (JSM-7100F; Jeol, Tokyo, Japan) (Fig. 1).

### CMA3 Peptide Synthesis and Purification

The CMA3 peptide (KWKLKKHIGIGKHFLSAKKF-NH2) was synthesized as previously described [42] using the 9-fluorenylmethoxycarbonyl (Fmoc) solid-phase method on Rink amide 4-methyl benzhydrylamine resin (0.55 mmol/g; Novabiochem, Darmstadt, Germany) with a Liberty microwave peptide synthesizer (CEM Co., Matthews, NC, USA). To obtain N-terminal fluorescently labeled peptides, resin-bound peptides were treated with 20% (*v*/*v*) piperidine in dimethylformamide to remove the protective Fmoc group from the N-terminal amino acid residue. The peptides were then cleaved from the corresponding resins, precipitated with ether, and extracted. The resultant crude peptides were purified by reversed-phase preparative high-performance liquid chromatography (HPLC) on a Jupiter C_18_ column (250 × 21.2 mm, 15 μM, 300 Å) using a 0–60% acetonitrile gradient in water containing 0.05% trifluoroacetic acid. The purity of the extracted peptides (more than 95%) was then confirmed using analytical reversed-phase HPLC on a Jupiter proteo C_18_ column (250 × 4.6 mm, 90 Å, 4 μM). The molecular mass of the peptides was confirmed using a matrix-assisted laser desorption ionization mass spectrometer (MALDI II; Kratos Analytical, Inc., Spring Valley, NY, USA).

### Electrophysiology

A single heptameric α-hemolysin protein pore (α-HL) (Sigma-Aldrich, Germany) was allowed to self-assemble into an artificial lipid membrane composed of 1,2-diphytanoyl-sn-glycero-phosphocholine (Avanti Polar Lipids, Alabaster, AL, USA), reconstituted across a circular aperture with a diameter of ~150 μm punctured in a 25-μm-thick Teflon film (Goodfellow, Malvern, MA. USA), pre-treated with 1:10 hexadecane/pentane (HPLC-grade, Sigma-Aldrich, Germany), separating the two polycarbonate chambers (volumes of 1 mL) of the recording cell. The chambers were filled with a 0.5-M KCl solution buffered with 10 mM HEPES at pH = 7 and 5 mM MES at pH = 4, respectively. The *trans* (not grounded) chamber was filled with a ~1.2 × 10^8^ cfu/mL of bacterial suspension containing either *P. aeruginosa* or *E. coli*, obtained by rehydration of the lyophilized bacteria with the same electrolytic solution. Upon addition of ~1 μL from a monomeric stock solution of α-HL made in 0.5 M KCl on the *cis* (grounded) side of the membrane and continuous stirring for about 5 min, successful insertion of the heptameric α-HL nanopore occurred. The ion current mediated by the α-HL nanopore was recorded in the voltage-clamp mode with an Axopatch 200B (Molecular Devices, USA) patch-clamp amplifier, at the room temperature of ~22 °C. When the experiments were performed in the presence of antimicrobial peptides, CMA3 was added to the *trans* chamber at a bulk concentration of 20 μM and was allowed to interact with the bacterial membranes for ~10 min before starting the recordings. Amplified electric signals reflecting ion current through the α-HL nanopore were low-pass filtered at a corner frequency of 10 kHz. Data acquisition was performed with a NI PCI 6221, 16-bit acquisition board (National Instruments, USA) at a sampling frequency of 50 kHz, using a LabVIEW 8.20 (National Instruments, USA) virtual instrument. Numerical analysis and graphical representation of the acquired data were performed using Origin 6 (OriginLab, USA) and pClamp 6.03 (Axon Instruments, USA) software.

## Results and Discussion

**Fig. 2:**
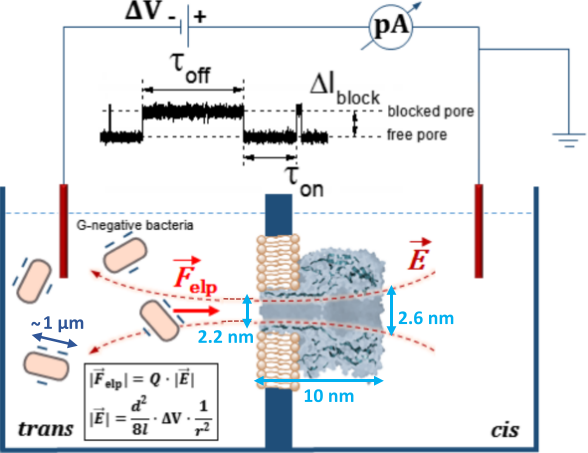
The working principle of the α-HL nanopore-based detection of bacteria at single-particle level. The negative electric potential on the *trans* side of a lipid membrane containing a single α-HL nanopore drives negatively charged bacteria into the nanopore. Within a spherical symmetry formalism, the absolute value of the electrophoretic force (*F*_elp_) acting on the bacteria is proportional to the negative electric charge on bacterial surface (*Q*) and the electric field (*E*) measured in bulk at a radial distance (*r*) from the pore, which emanates from the applied transmembrane potential (Δ*V*) [44]. In the inset formula, *d* and *l* represent the pore’s diameter and length. The bacteria-nanopore collisions at the lumen entrance of the α-HL determine brief obstructions of the nanopore’s permeating pathway, seen as reversible blockades of the ionic current, further measured with a sensitive current amplifier. The idealized trace inset allows us to define the main characteristics which are subsequently used to quantify the single bacteria-nanopore interactions, namely the extent of the ionic current blockade (Δ*I*_block_ = *I*_blocked pore_ − *I*_free pore_), average time of intervals measured between successive blockade events (*τ*_on_) and average time spent by the nanopore in the blocked state, following its interaction with a bacterium (*τ*_off_). Note that bacteria and the α-HL protein are not drawn to scale

We show the experimental principle of single-bacteria detection using the α-HL nanopore in Fig. 2. In the closed vicinity of a single nanopore inserted in a lipid membrane, whose electric potential on the *trans* side is negatively biased, the *trans*-added negatively charged bacteria are electrophoretically driven towards the lumen entrance of the protein by the electric field lines generated by the transmembrane potential. Despite the microscopic intricacies of the transport, which are different mainly due to the much larger hydrodynamic radius of bacteria—and therefore much diminished diffusion coefficient and mechanic deformability—as compared to individual peptides or DNA molecules, the actual electrophoretic coefficient of closely related bacteria is in the close order of magnitude of other small molecules as mentioned above (~10^−8^ m^2^V^−1^s^−1^) [43]. Therefore, we believe that at its core, the physical mechanism of the bacteria capture by a single α-HL nanopore may be similar (*vide infra*, also) to that encountered in the case of biomolecules [26, 33, 44]. Note that bacterial cells bear a net negative charge on their surface due to negative phosphate and carboxyl groups of LPS molecules [45, 46], and zeta potential values measured in low salt buffers, range from −22 to −55 mV for the *E. coli* [47] and from −11 to −42 mV, respectively, for *P. aeruginosa* cells [48, 49]. We conclude that by the virtue of physical principles explained in Fig. 2, a *trans*-negative transmembrane potential creates electric field lines extending from the bulk electrolyte into the entrance of the nanopore, enabling the electrophoretic funneling of bacteria towards the α-HL lumen entrance, and give rise to a temporary reduction in the ionic current through the nanopore, suggestive of bacterial cell detection at the single-particle level as depicted graphically in Fig. 2.

**Fig. 3:**
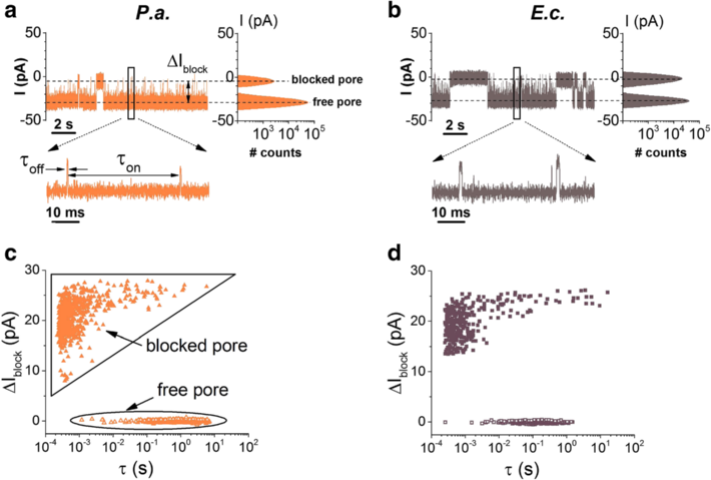
Demonstration of individual detection of *P. aeruginosa* (*P.a.*) and *E. coli* (*E.c.*) with the α-HL. Original current recordings showing reversible current blockage events induced by the association of *P.a.* (**a**) and *E.c.* (**b**), respectively, with the α-HL. The amplitude histograms showed on the right side indicate the nearly complete ionic flow obstruction through the nanopore by either bacterium. Selected time intervals characterizing the frequency (*τ*_on_) and duration (*τ*_off_) of bacteria-α-HL interactions are shown in the *inset* of **a. c**, **d** Scatter plots of current blockade versus dwell time of blockade events reflecting the *P. aeruginosa* (*P.a.*) and *E. coli* (*E.c.*) interaction with the α-HL. The distinct states of the nanopore, i.e., free and temporarily blocked by a bacterium, are denoted in **c**. The applied transmembrane potential was Δ*V* = −80 mV

In Fig. 3, we display typical recordings probing the reversible changes in the ionic current through a single α-HL nanopore, when either *P. aeruginosa* or *E. coli* bacterial cells were added on the negatively biased *trans* side of the membrane, at an optimal concentration in our experiments of 1.2 × 10^8^ cfu/mL. During the course of preliminary recordings carried out in the presence of less concentrated bacterial cells added in the electrolyte (~8 × 10^7^ cfu/mL), the scarcity of the bacteria-α-HL nanopore blockade events precluded a reliable statistical analysis (data not shown). Control experiments performed in the absence of bacteria, or in the presence of bacteria added on the *trans* side, but positively biased membranes, confirmed the absence of such blockade events (Additional file 1: Figure S1).

Given the experimental specifics of microbiology protocols used herein, e.g., the rehydration of bacterial cells, it cannot be excluded that anionic, inner constituents of bacteria or even anionic macromolecules from the bacterial membrane may get released in the buffer, interact with the negatively biased nanopore, and thus are prone to interfere with the blockade events shown in Fig. 3. The net concentration of such constituents is expected to be extremely low to be detected with the α-HL nanopore, given that the pool of bacteria they could come from is only in the order of 10^8^ cfu/mL. On the other hand, it should be recollected that within simplifying assumptions, the passage of a spherical analyte through a cylindrical nanopore generates a current block across the nanopore ΔI_block_, whose modulus is directly proportional to the conducting volume excluded by the analyte (δ), $\varDelta {I}_{\mathrm{block}}=\frac{\sigma_{\mathrm{buffer}}\varDelta V\delta }{l_p^2}$ (*σ*_buffer_ represents the conductivity of the buffer, Δ*V* is the transmembrane potential across the nanopore, and *l*_p_ is the nanopore’s length) [27]. From this volumetric perspective alone, this formula tells us that analytes with variable volumes (*δ*) entering the nanopore, as it would be the case of individual molecules or molecular oligomers detached from bacterial cells (*vide supra*), are expected to generate heterogeneous blockade events whose magnitudes (*ΔI*_block_) would span a wider range of values, proportional to their volume. As a direct example in this respect, in previous work we have shown that minute variations on the physical and chemical properties of the same analyte result in distinguishable values of the current block across the nanopore (Δ*I*_block_) [33], and this is one attribute which makes the nanopore approach so useful for single-molecule studies and identification of even closely related molecules.

In contrast, the all-points histogram presented in Fig. 3 and below reveal only two Gaussian peaks, assigned to the free and the α-HL blocked nanopore. All things considered, we conclude that blockade events detected in our experiments (Fig. 3 and below) reflect bacteria-α-HL reversible interactions.

**Fig. 4:**
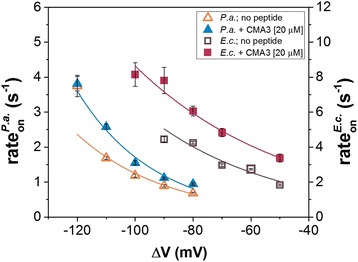
Capture propensity of *P. aeruginosa* (*P.a.*) and *E. coli* (*E.c.*) by α-HL, as a function of the applied voltage. Voltage dependence of the capture rate (rate_on_) of bacterial cells by the α-HL nanopore for *P.a.* and *E.c.* in the absence (*open triangles* and *rectangles*, respectively) and in the presence of a synthetic antimicrobial peptide (CMA3) added on the *trans* chamber at a bulk concentration of 20 μM (*closed triangles* and *rectangles*, respectively), at pH = 7

The current blockade signature through the α-HL nanopore held at Δ*V* = −80 mV, caused by bacterial cell-α-HL collisions (Fig. 3a, b) and seen as upwardly oriented current spikes from ~−28 ± 0.5 pA (open nanopore) to ~−3.8 ± 0.6 pA (blocked nanopore)) contain very short events (seen better in the zoomed-in insets) and longer ones. As indicated by scatter plots of points corresponding to the inter-event time distribution shown in Fig. 3c, d, the mean association time of *E. coli* to the α-HL, calculated from the time distribution of events belonging to the “free pore” distribution, is roughly one order of magnitude faster than that of *P. aeruginosa* (*τ*_on_^*E.c.*^ = 0.23 ± 0.004 s; *τ*_on_^*P.a.*^ = 1.47 ± 0.05 s). To confirm this further, the dynamics of the reversible association between bacterial cells and a single α-HL nanopore was investigated at different applied potentials, and we computed the reciprocal of the mean inter-event times (*τ*_on_), i.e., the bacteria-α-HL association rate (rate_on_). As shown in Fig. 4, rate_on_ for either type of bacterial cells increases exponentially with the applied voltage, and *E. coli* associates faster than *P. aeruginosa* to the α-HL within the range of the applied transmembrane potentials. For each applied voltage, the experiments were performed several times in order to probe the reproducibility. In Additional file 1: Figure S4, we show the additional analysis of dissociation times (*τ*_off_) of individual bacteria from the nanopore, which strengthen further the distinct kinetic pattern of such interactions depending upon the bacterial cells under investigation. Note however that unlike experiments done with macromolecules able to translocate across the nanopore (e.g., peptides, nucleic acids), bacteria are sterically excluded from such excursions, meaning that our reported dissociation times reflect solely bacteria unbinding from the nanopore to the same side, under thermal forces.

A relevant issue we tried to address next was to shed light on the dominant model at play, regarding the mechanism of bacterial cell-α-HL nanopore association. As it is known, one of the widely used models describing the thermodynamics and kinetic pathway of interactions taking place between individual molecules makes use of the transient complex formation during a chemical transformation (or molecular interaction, for that matter) and is being placed at the core of the classical transition-state theory [50]. In contrast, we investigate herein interactions taking place between macroscopic particles (i.e., bacterial cells) and individual proteins (i.e., the α-HL nanopore) rather than interactions taking place between individual molecules, and this may restrict the applicability of the classical transition-state theory. However, it is noteworthy that the α-HL nanopore is immobilized in the lipid membrane, thus exposing only a limited domain roughly consisting of the 2 nm in diameter opening of the α-HL’s β-barrel on the *trans* side (see cartoon representations in Figs. 2 and 6), to enable the capture of the *trans*-added bacterial cells. In other words, despite their ~μm range in diameter (Fig. 1), the bacterial cells association to the α-HL nanopore are mediated by molecular interactions taking place solely on a nanometer-scale surface patch on the incoming bacteria, which are being guided by the transmembrane potential towards the α-HL’s β-barrel opening on the *trans* side. These considerations allow us to recognize, among others (*vide infra*), the classical Kramers’ theory as a putative mechanism describing the association of bacteria with the nanopore.

Inspired from previous studies [33, 44, 51], the capture mechanism of bacterial cells investigated herein can be described either in the diffusion-limited regime, case in which the capture rate would be limited by the time required for the bacteria to arrive at the pore, or with the energy barrier model, in which the capture rate is described in the framework of the classical Kramers’ theory, according to which the delivery of the bacterial cells is necessary but not sufficient to result in binding to the pore’s mouth, as they need to acquire energy to overcome an energetic barrier for the binding to occur. The salient feature which distinguishes between the two models lies in the voltage dependence of the association rate, which is predicted to vary linearly with the applied voltage for the case of the diffusion-limited regime, whereas it displays an exponential increase with the applied voltage, for the energy barrier model. Thus, the results presented in Fig. 4 are consistent with an energy barrier model in which bacterial cells are delivered to the pore mouth multiple times before a successful, transient binding occurs. As it has previously been used to describe the entry of proteins into a nanopore [52], the voltage dependence of rate_on_, describing the association of both *E. coli* and *P. aeruginosa* with the α-HL, was fitted with an exponential curve consistent with the Van’t Hoff-Arrhenius law, expressed by the equation: ${\mathrm{rate}}_{\mathrm{on}}=A\cdot \mathrm{ex}p\left(\frac{q\varDelta V-{U}^{*}}{k_B{T}_m}\right)$ *,* where *q* is the effective electric charge at the surface of the bacterial cell which has to surpass the energy barrier U*, at the entrance of the α-HL pore, Δ*V* is the negatively applied transmembrane potential, *k*_*B*_ is the Boltzmann constant, and *T*_*m*_ is the absolute temperature of the environment. The constant *A* depends on the concentration of bacterial cells, the diffusion coefficient of bacteria, and the geometry of the protein pore [26], and given the close geometric resemblance of *E. coli* and *P. aeruginosa*, we consider it similar for the two types of bacteria to a first approximation. The above expression can be written as ${\mathrm{rate}}_{\mathrm{on}}={r}_0\cdot \exp \left(\frac{q\varDelta V}{k_B{T}_m}\right)$ where the constant term ${r}_0=A\cdot \exp \left(-\frac{U^{*}}{k_B{T}_m}\right)$ represents the association rate in the absence of the applied voltage. We note that among others, the energy barrier term (U*) contains lumped contributions related to electrostatic repulsive interactions manifested between the negatively charged bacterial cells and the lumen entrance of the α-HL nanopore, composed of 14 aspartic acids (D127 and D128) and seven lysines (K131) from the seven protein monomers, which at neutral pH assumes a net negative charge [53].

For the negatively charged surface of the bacteria, the electric charge *q* is equal to −*z*|*e*−|, where *z* represents the effective electric valence and *e* ^−^ is the elementary charge of the electron. The values for *r*_0_ and *z*, respectively, as determined from the fitted exponential curves, are summarized in Table 1. At least two important remarks should be made regarding this result: (i) although the net electric surface charge of bacteria is far larger, the fact that the effective electric charge contributing to bacteria-α-HL interactions is barely in the order of an elementary charge comes as no surprise, considering that the dimensions of bacterial cells are in the order of micrometers, while the α-HL nanopore opening dimensions are in the order of nanometers [16]. That is, the electric field created by the external applied voltage and extending out of the α-HL’s lumen into the *trans* chamber will act upon a very limited number of negative charges present on the bacterial surface, which are localized in the close proximity of the nanopore. Moreover, given that at a room temperature of *T*_m_ = 300 K, the Debye length *κ* ^−1^ for a KCl solution of 0.5 M ionic strength is approximately 0.4 nm $\left({k}^{-1}=\sqrt{\frac{\epsilon_r{\epsilon}_0{k}_B{T}_m}{2{\left|{e}^{-}\right|}^2{N}_AI1000}}\right)$, where *ε*_r_ and *ε*_0_ represent the relative permittivity of the electrolyte (*ε*_r_ ~ 70) and vacuum permittivity, respectively, *N*_A_ is Avogadro’s number, *e* ^−^ stands for the elementary charge, *k*_B_ is Boltzmann constant, *T*_m_ the absolute temperature, and *I* represents the ionic strength of electrolyte in molar units; a limited number of negative charges belonging to the extremities of the O-side chains of LPS which make up for the charge distribution on the bacterial surface [54, 55] will interact electrostatically with the charged ring at the *trans* opening of the α-HL nanopore. (ii) The effective electric charges contributing to bacteria-α-HL interactions were found to be lower for *E. coli* cells as compared to *P. aeruginosa*. This creates an apparent paradox, considering that the capture process of *E. coli* by the α-HL nanopore, which is governed in part by the electric attractive interactions between bacterial cells and the electric field lines at the entrance of the nanopore (Fig. 2), is higher as compared to *P. aeruginosa*. As we will also demonstrate below, we conclude that electrostatic repulsive interactions manifested between the negatively charged bacteria and the α-HL’s lumen entrance contribute to an increase in the free energy barrier needed to be overcome (U*, *vide supra*), as the bacteria proceeds along its pathway to the nanopore’s entrance, leading to its transient association to the α-HL. Thus, in qualitative terms, less charged bacteria (i.e., *E. coli*) will associate more easily with the α-HL nanopore. This is also confirmed by the fact that the constant term ${r}_0=A\cdot \exp \left(-\frac{U^{*}}{k_B{T}_m}\right)$ from the model used to characterize the dynamics of bacteria-α-HL interactions (*vide supra*) is almost one order of magnitude larger for *E. coli* than for *P. aeruginosa* cells (see Table 1).

**Table 1:** Values for the capture rate (*r*_0_) of *P. aeruginosa* (*P.a.*) and *E. coli* (*E.c.*) cells by the α-HL nanopore in the absence of the applied voltage, and the effective electric valence (quantified by *z*, see text) on the bacterial surface in contact with the α-HL’s lumen opening, estimated in the absence and presence of *trans*-added CMA3 peptide (20 μM) from the exponential fits shown in Fig. 4

	No peptide		+CMA3 (20 μM)	
	*P.a.*	*E.c.*	*P.a.* +CMA3	*E.c.* +CMA3
*r*_0_ (s^−1^)	0.04 ± 0.02	0.66 ± 0.17	0.05 ± 0.02	1.33 ± 0.14
*z*	0.95 ± 0.10	0.58 ± 0.09	0.82 ± 0.10	0.48 ± 0.04

In a recent paper [42], we demonstrated that a synthesized antimicrobial peptide termed CMA3, derived from two naturally occurring AMPs, cecropin A and magainin 2, showed antimicrobial activity against drug-resistant *E. coli* and *P. aeruginosa* strains used herein. A key step leading to microbe lysis by AMPs belonging to this class is represented by the binding and subsequent insertion of peptides into the bacterial membrane [56], driven by a combination of peptide–bacteria electrostatic and hydrophobic interactions. As a consequence, and as demonstrated previously by us and other authors, the cationic CMA3 peptide adsorption to the outer membrane containing negatively charged LPS progressively neutralizes the net negative charge on the bacterial surface, and this will be directly correlated to the amount of peptide bound to bacteria [42, 47, 57].

Based on these, we employed the CMA3 peptide as a putative molecular recognition element and sought to interrogate basic biophysical aspects on how the specificity of antimicrobial peptide–bacteria interactions lead to a measurable response, when detecting the two types of bacteria tested herein with the α-HL nanopore.

**Fig. 5:**
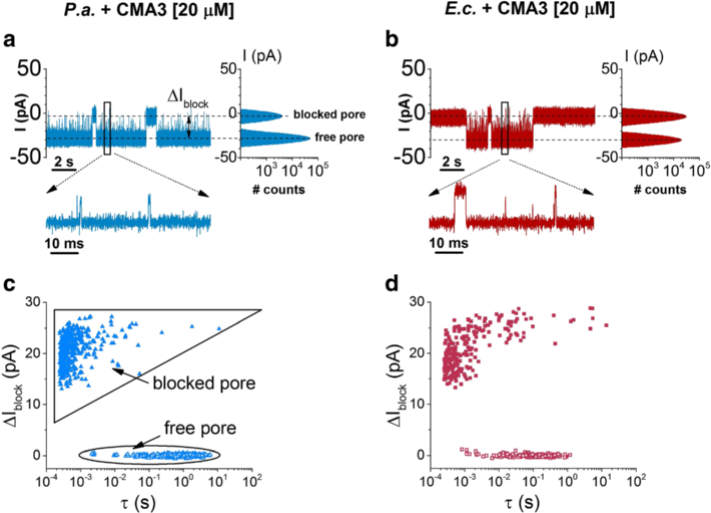
Original current recordings showing reversible current blockage events induced by the association of *P. aeruginosa* (*P.a.*) (**a**) and *E. coli* (*E.c.*) (**b**), respectively, with the α-HL, in the presence of a synthetic antimicrobial peptide (CMA3) added on the *trans* chamber at a bulk concentration of 20 μM, at Δ*V* = −80 mV. In **c** and **d**, we show the corresponding scatter plots of current blockade versus dwell time of blockade events reflecting the *P.a.* and *E.c.* interaction with the α-HL pore in the presence of the peptide. The distinct states of the nanopore, i.e., free and temporarily blocked by a bacterium, are denoted in **c**

In Fig. 5a, b, we show selected recordings of reversible ion current blockades through the α-HL nanopore caused by the association of either *P. aeruginosa* or *E. coli* cells with the nanopore held at Δ*V* = −80 mV, in the presence of CMA3 peptide (20 μM) added on the *trans* side of the membrane. Note that as a consequence of the net positive charge (+8|e^−^|) on CMA3 at neutral pH, individual peptide-nanopore interactions are excluded at negative potentials. Thus, the events seen are indicative solely of bacteria-nanopore collisions. To further substantiate this, we show in Additional file 1: Figures S2 and S3 ion current traces recorded in control experiments (i.e., the absence of bacterial cells), demonstrating the absence and, respectively, presence of CMA3-α-HL reversible interactions, as seen at negative and positive transmembrane potentials held across the nanopore, similar in absolute values to those used herein.

As argued above, the adsorption of the cationic CMA3 to either bacterial strain will decrease the net value of the negative charge on their outer membrane, and this would tend to lower the electrophoretically driven capture rate of bacteria by the α-HL nanopore (see also Fig. 1). Apparently counterintuitive, the scatter plot analysis indicates a slight, yet visible, decrease in the association time of either bacterium at the α-HL’s entrance as compared to the case of no peptide added, which would correspond to an increase in the capture rate of bacteria by the nanopore (Fig. 5c, d). For a better quantitative evaluation, the dynamics of the reversible association between bacterial cells and a single α-HL nanopore, taking place in the absence or presence of the CMA3 peptide, was calculated from the inverse of the average association time (*τ*_on_) of bacterial cells to the pore, as the capture rate (rate_on_) of bacteria by the nanopore, and shown to be voltage dependent (Fig. 4).

Data points shown in Fig. 4 suggest that an increase in the transmembrane potential leads to correspondingly lower association times of either bacteria strain to the α-HL, which is expected due to the increase in the electrophoretic force acting on bacteria, and the association process proceeds faster for the *E.c.* than *P.a.* bacteria. By contrast, at a given value of the transmembrane potential, a CMA3 peptide-complexed bacteria—which would display a net surface charge deficit—associates *faster* with the α-HL nanopore at the lumen entrance, while the overall voltage-dependent association follows the same tendency as for the peptide-free bacteria. Thus, within the range of transmembrane potentials which allows the optimal recording of the two types of bacterial cell interaction with the α-HL in the absence and presence of the CMA3 peptide, Δ*V* = −80 mV ÷ −110 mV for *P. aeruginosa* and Δ*V* = −50 mV ÷ −90 mV for *E. coli*, the addition of 20 μM CMA3 decreased the average times measured between successive capture events of bacterial cells (*τ*_on_) with 26.5 ± 6.2% for *P. aeruginosa* and 39.3 ± 6.9% for *E. coli*, respectively.

**Fig. 6:**
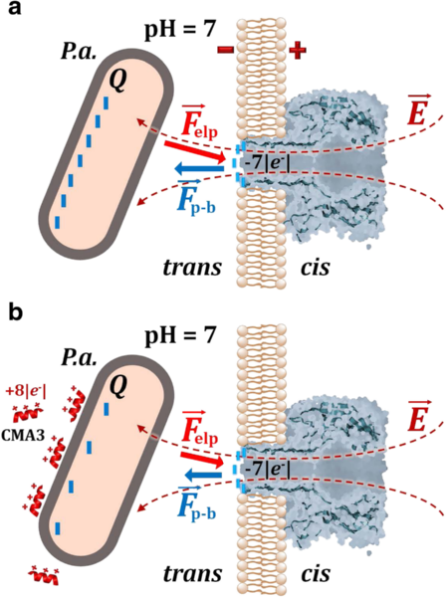
Cartoon representation (not drawn to the scale) reflecting the putative role played by the CMA3 peptide adsorption to *P.a.*, on the bacterial cell capture process. As compared to the control case (no peptide, **a**), the cationic CMA3 peptide (charge = + 8|*e* ^−^| at pH = 7) adsorption to *P.a.* (**b**) determines a net decrease on the bacterial surface charge on the outer membrane (Q), at pH = 7. The charge reduction on bacterial surface leads to an augmented association rate of the *P.a.*-α-HL interactions, resulting from the reduced magnitude of electrostatic repulsive forces manifested between the negatively charged bacteria and the lumen entrance of the α-HL (F_p-b_), which over-compensates for the reduced electrophoretic force (*F*_elp_) acting on the bacterium (see text). Around neutral pH, the net electric charge present at the entrance of α-HL’s lumen is ~ − 7|*e* ^−^|

As presented above, to explain this apparent paradox, we note that the entrance of the α-HL’s β-barrel lumen, pointing towards the *trans* side, is negatively charged at neutral pH [53]. On a simplified yet realistic framework, we assume that the primary site of bacteria interaction with the α-HL nanopore, leading to its transient capture and the obstruction of ions passage across the nanopore, occurs at the *trans* mouth of the α-HL’s lumen. Thus, a negative charge distribution on this region reflects as an electrostatic barrier for the electrophoretically driven, negatively charged bacteria towards the α-HL (Fig. 6). In previous work, we demonstrated the crucial role played by such interactions for the capture of peptides by the α-HL [58].

We conclude that a decrease on the negative charge present on the bacteria surface, as induced in the present case by the membrane-adsorbed CMA3 peptide, will contribute both to a decrease in the electrostatic component of the energy height a bacterium must surpass to attach to the pore from the *trans* side, and a build-up of bacterial cells concentration at the barrier entrance via diminished bacteria-α-HL repulsive electrostatic interactions [44], and this would explain fairly well the data presented in Fig. 5. In quantitative terms, and by employing a similar formalism as above, the effective electric charge on the surface of the bacterial cells and the constant term ${r}_0=A\cdot \exp \left(-\frac{U^{*}}{k_B{T}_m}\right)$ from the model used to characterize the dynamics of bacteria-α-HL interactions in the presence of the CMA3 peptide were calculated from the nonlinear fit of data shown in Fig. 4 and are displayed in Table 1.

To further probe the relevance of electrostatic interactions manifested between the bacterial cells and the α-HL lumen opening, we conducted experiments at a pH = 4 set in both chambers of the bilayer setup. Under such conditions, the 14 aspartic acids (Asp127 and Asp128 in each of the heptamers of the α-HL pore assembly, pK_a_ values ~3.9) will be partially protonated, thus leading to a dramatic decrease in the negative charge at the entrance of the protein pore. Note that the bacterial species used herein are negatively charged over a wide range of pH values. However, at acidic pH’s and depending upon the particular bacterial cells, the net surface charge of bacterial cells’ outer membrane may be slightly diminished as compared to neutral pH, as reported for *E. coli* cells [59]. The rationale was that if the bacteria-α-HL collisions were controlled, among others, by the repulsive electrostatic interactions manifested at the negatively charged lumen entrance of the nanopore, a partial neutralization of these charges would facilitate bacteria-α-HL association.

**Fig. 7:**
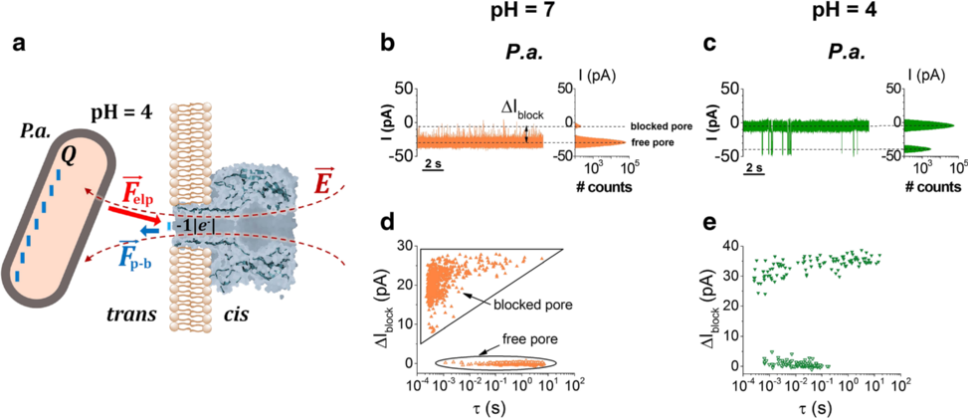
*P. aeruginosa* (*P.a.*) interaction with the α-HL at neutral and acidic pH’s. **a** At pH = 4, the partial depletion of the net negative charge at the lumen entrance of the α-HL (~ − 1|*e* ^−^|) results on a reduced electrostatic repulsive forces manifested between the negatively charged bacteria and the lumen entrance of the α-HL (*F*_p-b_) as compared to neutral pH (see also Fig. 6). In **b** and **c**, we show representative traces displaying the current fluctuations entailed by *P.a.* occluding the α-HL at pH = 7 and pH = 4, respectively. The applied transmembrane potential was Δ*V* = −80 mV. In **d** and **e**, we represent the events scatter plot of current blockade (Δ*I*_block_) versus dwell time of the free and blocked pore, for traces recorded at pH = 7 and pH = 4

This expectation is fully consistent with experimental observations, which involved only *P.a.* for this objective, showing an almost two-order of magnitude decrease in the bacteria-α-HL mean association time at pH = 4, as compared to experiments carried out at pH = 7 (Fig. 7, data corresponding to the distribution of “free pore” events). In addition, this observation suggests that acidic electrolytes may present better opportunities to record bacteria-α-HL blockades, and in conjunction with alternative ways aimed at enhancing the bacteria capture rate, e.g., the use of salt gradients across the nanopore [28], this may be useful in forthcoming studies to improve the detection limit of our approach.

As a cautionary note, we remind that previous work has revealed the presence of a so-called gating dynamics of the α-HL nanopore at acidic pH, which could interfere with the stochastic changes in the current across the nanopore generated by the bacterial cell-nanopore interactions described herein [60]. As we show herein by our control experiments (Additional file 1: Figure S5), such intrinsic, stochastic changes in the ion current through the protein at low pH have a distinct appearance in terms of frequency of occurrence and duration as compared to the blockade events caused by individual bacteria colliding to the nanopore, so they could be reliably excluded from our kinetic analysis. Moreover, prevalent intrinsic gating events on the a-HL nanopore were seen only at higher transmembrane potentials as compared to those used in this study (Additional file 1: Figure S5, panel d). The scatter plot analysis shown in Fig. 7d, e, illustrates that the mean dissociation time of the *P.a.*-α-HL complex at pH = 4 (*τ*_off_ = 1.4 s; data corresponding to the “blocked pore”) is almost two orders of magnitude larger as compared to pH = 7 (*τ*_off_ = 0.02 s). This may be also a consequence of the reduced repulsive interactions manifested between the *P.a.* and the α-HL, which would facilitate the bacterium residence at the α-HL’s lumen entrance in acidic pH’s as compared to neutral ones.

**Fig. 8:**
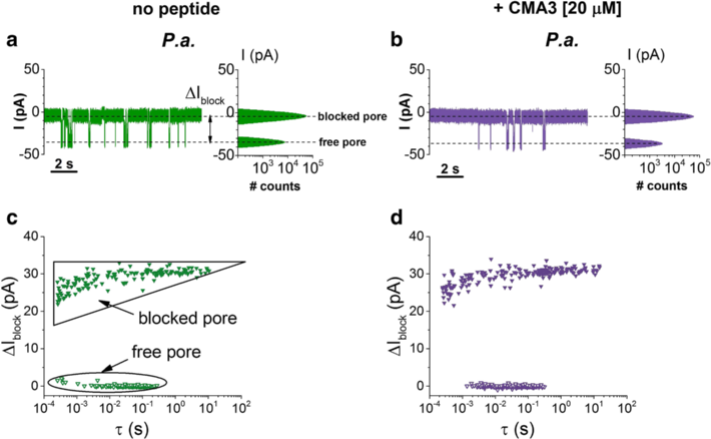
Electrical characterization of *P. aeruginosa* (*P.a.*) interaction with the α-HL at acidic pH. Comparison of *P.a.*-induced ion current blockades through the α-HL recorded at pH = 4 and Δ*V* = −70 mV, in the absence (**a**) and presence of the *trans*-added CMA3 peptide (**b**). In **c** and **d**, we show the events scatter plots of Δ*I*_block_ and dwell times of the free and *P.a.*-blocked α-HL corresponding to the representative traces shown in **a** and **b**. The comparison of dwell times distribution corresponding to the “free pore” reveals the virtual absence of the CMA3 peptide effect on bacterial cells association to the nanopore

**Fig. 9:**
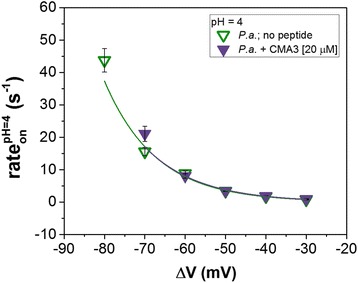
CMA3 peptide influence on the voltage dependence of *P.a.* capture by the α-HL nanopore at acidic pH. Data points represent rate_on_ values versus Δ*V* measured in the absence (*open triangles*) and presence of 20 μM CMA3 peptide (*full triangles*), at pH = 4

Interestingly, the further addition of the CMA3 peptide on the electrolyte solution at pH = 4 does very little to the association kinetics of the *P.a.* to the α-HL as compared to neutral pH (Fig. 8), and this tendency is preserved at any applied transmembrane potential (Fig. 9). Again, this is counterintuitive since the cationic CMA3 association to the bacterium surface is expected to diminish the net value of negative charge density on the surface, and thus further augment *P.a.*-α-HL association process. A possible explanation for this phenomenon may have to do with the decreased ability of such synthetic antimicrobial peptides to bind to the bacterium surface at low pH’s, meaning that the overall surface charge remains largely un-changed when the peptide is added to the buffer solution. This hypothesis remains to be tested further.

## Conclusions

Within the scope of this work, we have demonstrated the first proof of concept of a nanopore system based upon the α-HL protein to detect *P. aeruginosa* or *E. coli* presence in aqueous buffers. We capitalized on the ability of a selected antimicrobial peptide to interact specifically with and alter to a different extent the outer membrane surface charge density of bacterial cells and illustrated the impact of altering the surface charge of the bacterial cells on their detection by the α-HL nanopore. Based on these findings, we suggest the potential of using the α-HL nanopore in conjunction with molecular probes such as more specific AMP’s, or bacteria-specific, multivalent functionalized nanostructured substrates [61], to enable detection of bacteria in real-life samples. This is achievable by exploiting such analyte’s ability to target and modify selectively the outer membrane charge density of pathogens, whose electrophoretic-mediated interaction with the nanopore will generate blockade events of the current across the α-HL with distinct kinetic patterns than those of other particles present in the buffer.

It should be noted that despite its appeal in terms of development simplicity, one of the shortcomings of the approach reported herein towards detecting with specificity bacterial cells lies in the difficulty of particle discrimination based solely on their physical volume. Namely, for the case of large particles comparable in size with the bacteria used herein, which are precluded from entering the inner volume of the α-HL, identification based solely on their size would be almost impossible as the current blockade extent across the nanopore would be rather similar. The electrophoretic mobility and net charge on the particles are still expected to influence the kinetics of such blockade events, as our data demonstrate that charge alteration of the bacterial surface leads to a distinct kinetic signature on bacteria-nanopore reversible interactions. As an alternative strategy for perspective, we propose that tailored peptides or specific antibodies to target bacterial surface more specifically, as to discern between Gram-negative and Gram-positive bacteria or even between pathogenic and nonpathogenic cells, could be covalently attached at the α-HL mouth via flexible linkers with optimal length and, at the same time, remain free to explore the nearby electrolyte. Thereupon, depending on the biorecognition probe(s) that is (are) hybridized to the nanopore, and based upon the principles revealed herein, such a construct might be used for the real-time, multiplex, and parallel detection of an extended spectrum of bacterial pathogens.

## Additional file

Additional file 1: Contains control experiments performed in the absence and presence of bacteria, added on the *trans* side, at positively biased membranes, control experiments performed at pH = 4 in the absence of bacteria, at negatively biased membranes, and control experiments performed in the presence of *trans*-added CMA3 peptides in the absence of bacterial cells, at negative and positive transmembrane potentials held across the nanopore. (DOCX 1419 kb)

## References

[1] Africa Check. https://africacheck.org/factsheets/factsheet-the-leading-causes-of-death-in-africa. Accessed 28 Apr 2016

[2] Sandhya S, Chen W, Mulchandani A (2008). Molecular beacons: a real-time polymerase chain reaction assay for detecting Escherichia coli from fresh produce and water. Anal Chim Acta.

[3] Klaschik S, Lehman LE, Gebel J, Book M, Steinhagen FW, Hoeft A, Stuber F (2011). Species-specific probes and real-time PCR as a tool for fast detection and differentiation of 15 bacteria relevant in intensive care medicine. World J Microbiol Biotechnol.

[4] Boyacı İH, Agauilar ZP, Hossain M, Halsall HB, Seliskar CJ, Heinman WR (2005). Amperometric determination of live Escherichia coli using antibody-coated paramagnetic beads. Anal Bioanal Chem.

[5] Zhu P, Shelton DR, Li S, Adams DL, Karns JS, Amstutz P, Tang CM (2011). Detection of E. coli O157:H7 by immunomagnetic separation coupled with fluorescence immunoassay. Biosens Bioelectron.

[6] Majumdar T, Chakraborty R, Raychaudhuri U (2013). Development of PEI-GA modified antibody based sensor for the detection of S. aureus in food samples. Food Biosci.

[7] Eisenstadt J, Washington JA, Mobley HL, Warren JW (1996). Diagnostic microbiology for bacteria and yeasts causing urinary tract infection. Urinary Tract Infections: Molecular Pathogenesis and Clinical Management.

[8] Puchberger-Enengl D, Podszun S, Heinz H, Hermann C, Vulto P, Urban GA (2011). Microfluidic concentration of bacteria by on-chip electrophoresis. Biomicrofluidics.

[9] Schwartz O, Bercovici M (2014). Microfluidic assay for continuous bacteria detection using antimicrobial peptides and isotachophoresis. Anal Chem.

[10] Mannoor MS, Zhang S, Link AJ, McAlpine MC (2010). Electrical detection of pathogenic bacteria via immobilized antimicrobial peptides. Proc Natl Acad Sci U S A.

[11] Lapizco-Encinas BH, Simmons BA, Cummings EB, Fintschenko Y (2004). Dielectrophoretic concentration and separation of live and dead bacteria in an array of insulators. Anal Chem.

[12] Yamada K, Kim CT, Kim JH, Chung JH, Lee HG, Jun S (2014). Single walled carbon nanotube-based junction biosensor for detection of Escherichia coli. PLoS One.

[13] Kang DK, Ali MM, Zhang K, Huang SS, Peterson E, Digman MA, Gratton E, Zhao W (2014). Rapid detection of single bacteria in unprocessed blood using Integrated Comprehensive Droplet Digital Detection. Nat Commun.

[14] Seo SH, Lee YR, Jeon JH, Hwang YP, Park PG, Ahn DR, Han KC, Rhie GE, Hong KJ (2015). Highly sensitive detection of a bio-threat pathogen by gold nanoparticle-based oligonucleotide-linked immunosorbent assay. Biosens Bioelectron.

[15] Majd S, Yusko EC, Billeh YN, Macrae MX, Yang J, Mayer M (2010). Applications of biological pores in nanomedicine, sensing, and nanoelectronics. Curr Opin Biotechnol.

[16] Gu LQ, Shim JW (2010). Single molecule sensing by nanopores and nanopore devices. Analyst.

[17] Kasianowicz JJ, Balijepalli AK, Ettedgui J, Forstater JH, Wang H, Zhang H, Robertson JW (2016). Analytical applications for pore-forming proteins. Biochim Biophys Acta.

[18] Bayley H, Cremer PS (2001). Stochastic sensors inspired by biology. Nature.

[19] Howorka S, Siwy Z (2009). Nanopore analytics: sensing of single molecules. Chem Soc Rev.

[20] Kasianowicz JJ, Brandin E, Branton D, Deamer DW (1996). Characterization of individual polynucleotide molecules using a membrane channel. Proc Natl Acad Sci U S A.

[21] Clarke J, Wu HC, Jayasinghe L, Patel A, Reid S, Bayley H (2009). Continuous base identification for single-molecule nanopore DNA sequencing. Nat Nanotechnol.

[22] Aksimentiev A (2010). Deciphering ionic current signatures of DNA transport through a nanopore. Nanoscale.

[23] Movileanu L, Schmittschmitt JP, Scholtz JM, Bayley H (2005). Interactions of peptides with a protein pore. Biophys J.

[24] Asandei A, Apetrei A, Park Y, Hahm KS, Luchian T (2011). Investigation of single-molecule kinetics mediated by weak hydrogen bonds within a biological nanopore. Langmuir.

[25] Apetrei A, Asandei A, Park Y, Hahm KS, Winterhalter M, Luchian T (2010). Unimolecular study of the interaction between the outer membrane protein OmpF from E. coli and an analogue of the HP (2-20) antimicrobial peptide. J Bioenerg Biomembr.

[26] Oukhaled A, Bacri L, Pastoriza-Gallego M, Betton JM, Pelta J (2012). Sensing proteins through nanopores: fundamental to applications. ACS Chem Biol.

[27] Talaga DS, Li J (2009). Single-molecule protein unfolding in solid state nanopores. J Am Chem Soc.

[28] Mereuta L, Asandei A, Seo C, Park Y, Luchian T (2014). Quantitative understanding of pH- and salt-mediated conformational folding of histidine-containing, β-hairpin-like peptides, through single-molecule probing with protein nanopores. ACS Appl Mater Interfaces.

[29] Asandei A, Mereuta L, Luchian T (2011). The kinetics of ampicillin complexation by γ-cyclodextrins. A single molecule approach. J Phys Chem B.

[30] Asandei A, Apetrei A, Luchian T (2011). Uni‐molecular detection and quantification of selected β‐lactam antibiotics with a hybrid α‐hemolysin protein pore. J Mol Recognit.

[31] Boersma J, Brain KL, Bayley H (2012). Real-time stochastic detection of multiple neurotransmitters with a protein nanopore. ACS Nano.

[32] Mereuta L, Schiopu I, Asandei A, Park Y, Hahm KS, Luchian T (2012). Protein nanopore-based, single-molecule exploration of copper binding to an antimicrobial-derived, histidine-containing chimera peptide. Langmuir.

[33] Asandei A, Schiopu I, Iftemi S, Mereuta L, Luchian T (2013). Investigation of Cu2+ binding to human and rat amyloid fragments Aβ (1-16) with a protein nanopore. Langmuir.

[34] Schiopu I, Iftemi S, Luchian T (2015). Nanopore investigation of the stereoselective interactions between Cu2+ and d, l-histidine amino acids engineered into an amyloidic fragment analogue. Langmuir.

[35] Wang G, Wang L, Han Y, Zhou S, Guan X (2014). Nanopore detection of copper ions using a polyhistidine probe. Biosens Bioelectron.

[36] Camerino DC, Desaphy JF (2010). Grand challenge for ion channels: an underexploited resource for therapeutics. Front Pharmacol.

[37] Wang Y, Gu L (2015). Biomedical diagnosis perspective of epigenetic detections using alpha-hemolysin nanopore. AIMS Mat Sci.

[38] Liu A, Zhao Q, Guan X (2010). Stochastic nanopore sensors for the detection of terrorist agents: current status and challenges. Anal Chim Acta.

[39] Darvish A, Goyal G, Kim M, Southern SO, Rodriguez-Chavez IR, Gärtner C, Stallings JD (2015). Sensing, capturing, and interrogation of single virus particles with solid state nanopores. Advances in global health through sensing technologies.

[40] Wu H, Chen Y, Zhoy Q, Wang R, Xia B, Ma D, Luo K, Liu Q (2016). Translocation of rigid rod-shaped virus through various solid-state nanopores. Anal Chem.

[41] McMuller A, de Haan HW, Tang JX, Stein D (2014). Stiff filamentous virus translocations through solid-state nanopores. Nat Commun.

[42] Lee JK, Seo CH, Luchian T, Park Y (2015). Antimicrobial peptide CMA3 derived from the CA-MA hybrid peptide: antibacterial and anti-inflammatory activities with low cytotoxicity and mechanism of action in Escherichia coli. Antimicrob Agents Chemother.

[43] van der Mei HC, Busscher HJ (2001). Electrophoretic mobility distributions of single-strain microbial populations. Appl Environ Mocrobiol.

[44] Grosberg AY, Rabin Y (2010). DNA capture into a nanopore: interplay of diffusion and electrohydrodynamics. J Chem Phys.

[45] Rietschel ET, Kirikae T, Schade FU, Mamat U, Schmidt G, Loppnow H, Ulmer AJ, Zähringer U, Seydel U, Di Padova F, Schreier M, Brade H (1994). Bacterial endotoxin: molecular relationships of structure to activity and function. FASEB J.

[46] Nikaido H (2003). Molecular basis of bacterial outer membrane permeability revisited. Microbiol Mol Biol Rev.

[47] Alves CS, Melo MN, Franquelim HG, Ferre R, Planas M, Feliu L, Bardaji E, Kowalczyk W, Andreu D, Santos NC, Fernandes MX, Castanho MA (2010). Escherichia coli cell surface perturbation and disruption induced by antimicrobial peptides BP100 and pepR. J Biol Chem.

[48] de Kerchove AJ, Elimelech M (2007). Impact of alginate conditioning film on deposition kinetics of motile and nonmotile Pseudomonas aeruginosa strains. Appl Environ Microbiol.

[49] Loughlin MF, Jones MV, Lambert PA (2002). Pseudomonas aeruginosa cells adapted to benzalkonium chloride show resistance to other membrane-active agents but not to clinically relevant antibiotics. J Antimicrob Chemother.

[50] Schreiber G, Haran G, Zhou HX (2009). Fundamental aspects of protein-protein association kinetics. Chem Rev.

[51] Wanunu M, Morrison W, Rabin Y, Grosberg AY, Meller A (2010). Electrostatic focusing of unlabelled DNA into nanoscale pores using a salt gradient. Nat Nanotechnol.

[52] Pastoriza-Gallego M, Rabah L, Gibrat G, Thiebot B, Van der Goot FG, Auvray L, Betton JM, Pelta J (2011). Dynamics of unfolded protein transport through an aerolysin pore. J Am Chem Soc.

[53] Wong CTA, Muthukumar M (2010). Polymer translocation through α-hemolysin pore with tunable polymer-pore electrostatic interaction. J Chem Phys.

[54] Poortinga AT, Bos R, Norde W, Busscher HJ (2002). Electric double layer interactions in bacterial adhesion to surfaces. Surf Sci Rep.

[55] King JD, Kocíncová D, Westman EL, Lam JS (2009). Review: Lipopolysaccharide biosynthesis in Pseudomonas aeruginosa. Innate Immun.

[56] Shin SY, Kang JH, Kim Y, Kim KL, Hahm KS (2000). Effects of the hinge region of cecropin A(1-8)-magainin 2(1-12), a synthetic antimicrobial peptide, on liposomes, bacterial and tumor cells. Biochim Biophys Acta.

[57] Freire JM, Domingues MM, Matos J, Melo MN, Veiga AS, Santos NC, Castanho MA (2011). Using zeta-potential measurements to quantify peptide partition to lipid membranes. Eur Biophys J.

[58] Asandei A, Chinappi M, Kang HK, Seo CH, Mereuta L, Park Y, Luchian T (2015). Acidity-mediated, electrostatic tuning of asymmetrically charged peptides interactions with protein nanopores. ACS Appl Mater Interfaces.

[59] Martinez RE, Smith DS, Kulczycki E, Ferris FG (2002). Determination of intrinsic bacterial surface acidity constants using a donnan shell model and a continuous pK(a) distribution method. J Colloid Interface Sci.

[60] Kasianowicz JJ, Bezrukov SM (1995). Protonation dynamics of the alpha-toxin ion channel from spectral analysis of pH-dependent current fluctuations. Biophys J.

[61] Pillai PP, Kowalczyk B, Kandere-Grzybowska K, Borkowska M, Grzybowski BA (2016). Engineering Gram selectivity of mixed-charge gold nanoparticles by tuning the balance of surface charges. Angew Chem.

