# Supplementary material for: A Protein Nanopore-Based Approach for Bacteria Sensing
Source: Nanoscale Res Lett. 2016 Nov 15;11:501. doi: 10.1186/s11671-016-1715-z (PMC5110462; doi:10.1186/s11671-016-1715-z)
Supplement: Additional file 1: — Contains control experiments performed in the absence and presence of bacteria, added on the trans side, at positively biased membranes, control experiments performed at pH = 4 in the absence of bacteria, at negatively biased membranes, and control experiments performed in the presence of trans-added CMA3 peptides in the absence of bacterial cells, at negative and positive transmembrane potentials held across the nanopore. (DOCX 1419 kb) [file 11671_2016_1715_MOESM1_ESM.docx]

**Supporting Information**

**A Protein Nanopore-Based Approach for Bacteria Sensing**

Aurelia Apetrei^1,#^, Andrei Ciuca^1,#^, Jong-kook Lee^2^, Chang Ho Seo^3^, Yoonkyung Park^3,*^

& Tudor Luchian^1,*^

^1^ Department of Physics, Alexandru I. Cuza University, Iasi, Romania.

^2^ Research Center for Proteineous Materials, Chosun University, Gwangju, South Korea

^3^ Department of Bioinformatics, Kongju National University, Kongju, South Korea.

# These authors contributed equally to this work.

*Corresponding authors ([y_k_park@chosun.ac.kr](mailto:y_k_park@chosun.ac.kr) and [luchian@uaic.ro](mailto:luchian@uaic.ro))


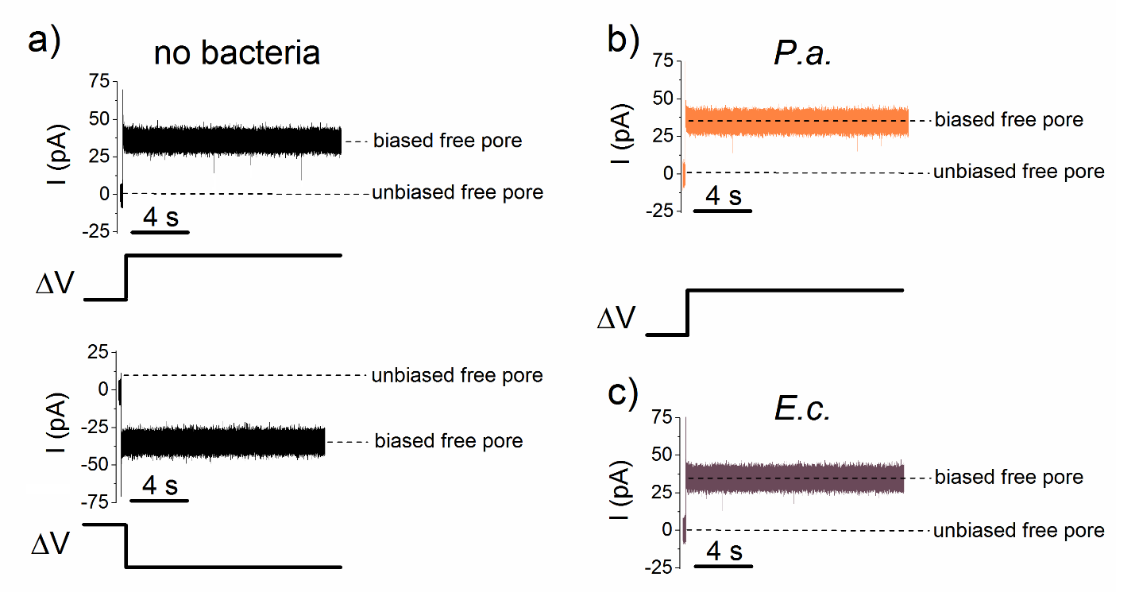


**Figure S1.** Panel (a) illustrates original current recordings through the α-HL protein nanopore in the absence of bacteria, at ΔV = +80 mV (upper graph) and -80 mV (lower graph), demonstrating the absence of current blockade events. In the presence of bacteria (*Pseudomonas aeruginosa* (*P.a.*) or *Escherichia coli* (*E.c.*)) added on the *trans* side, positively biased nanopores (ΔV = +80 mV) preclude their interaction with the bacterial cells, illustrated by the absence of blockade events (panels b and c). The recordings were performed at pH = 7, at a concentration of 1.2x10^8^ cfu/ml for either bacteria.


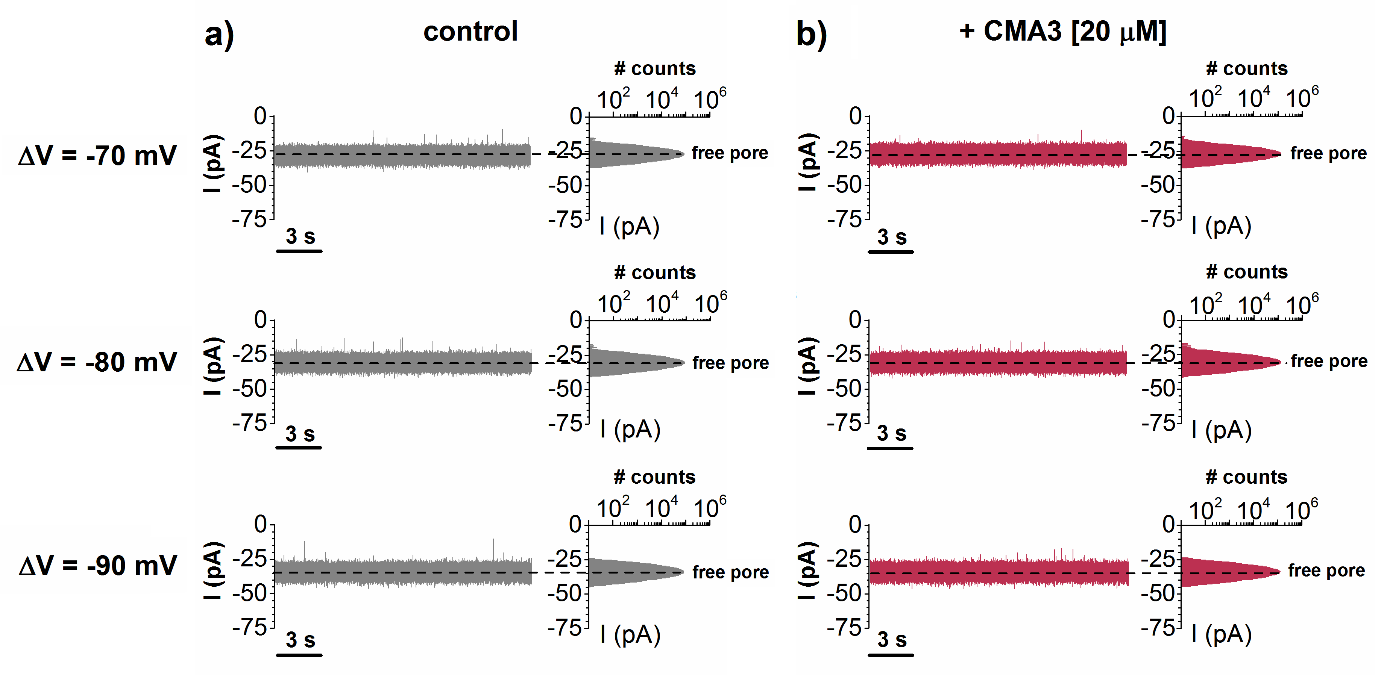


**Figure S2.** Panel (a) illustrates original current recordings through the α-HL protein pore in the absence of peptide and bacteria, at ΔV = -70 mV, -80 mV and -90 mV, respectively. Panel (b) shows selected current recordings through α-HL in the presence of 20 µM CMA3 peptide added on the *trans* side, in conditions of negatively biased membranes (ΔV = -70 mV, -80 mV, and -90 mV, respectively), demonstrating the lack of CMA3-α-HL interactions through the absence of specific blockade events. The corresponding amplitude histograms shown, indicate the value of the ion current through the free α-HL. All recordings were performed at pH = 7.


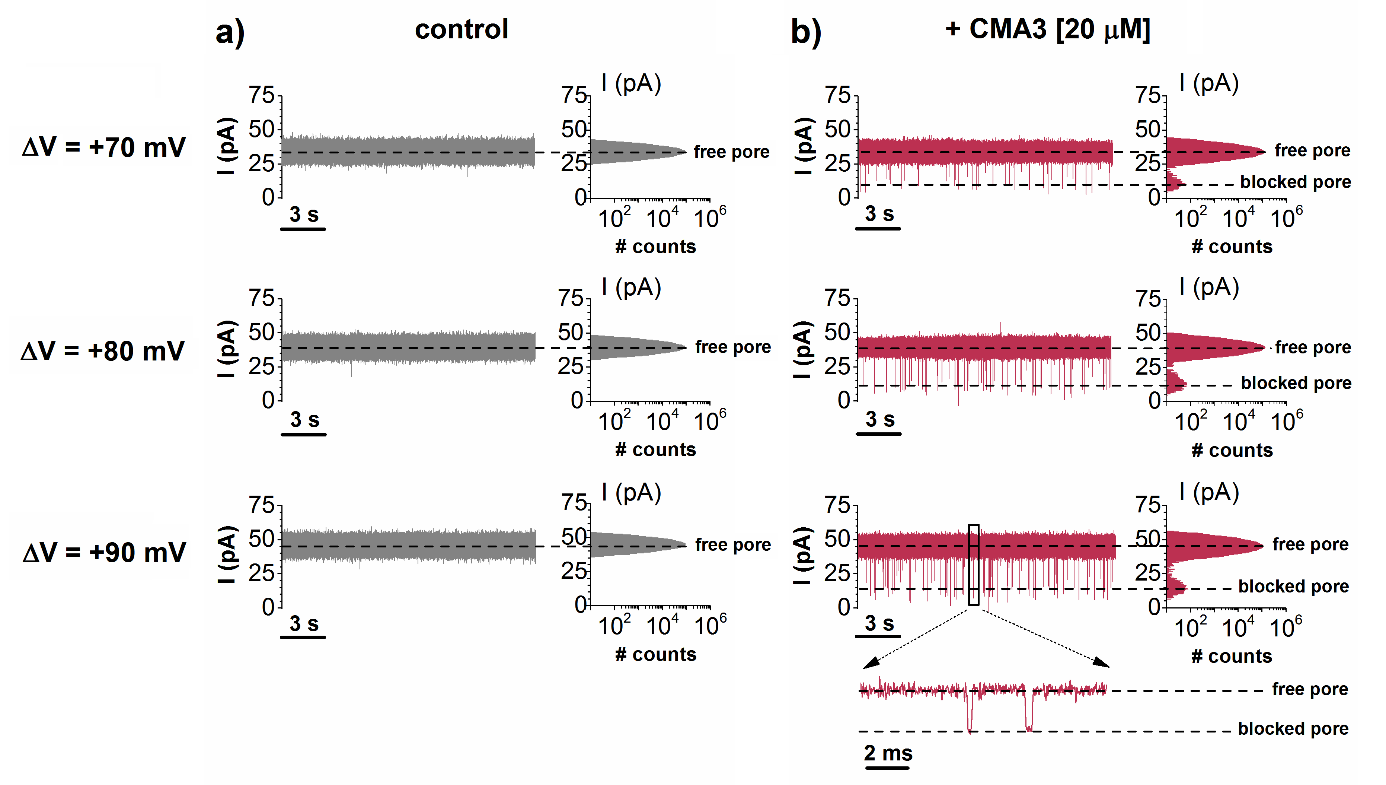


**Figure S3.** Panel (a) illustrates original current recordings through the α-HL protein pore in the absence of peptide and bacteria, in conditions of positively biased membranes (ΔV = +70 mV, +80 mV, and +90 mV, respectively). Panel (b) shows current recordings through α-HL in the presence of 20 µM CMA3 peptide added on the *trans* side, in conditions of positively biased membranes (ΔV = +70 mV, +80 mV, and +90 mV, respectively), revealing randomly occurring blockade events which reflect the reversible interactions between CMA3 and the α-HL protein pore, under the influence of the applied electric field. The distinct states of the nanopore, i.e. free and respectively reversibly blocked by a single peptide, are highlighted in the corresponding amplitude histograms. Recordings were performed at pH = 7.


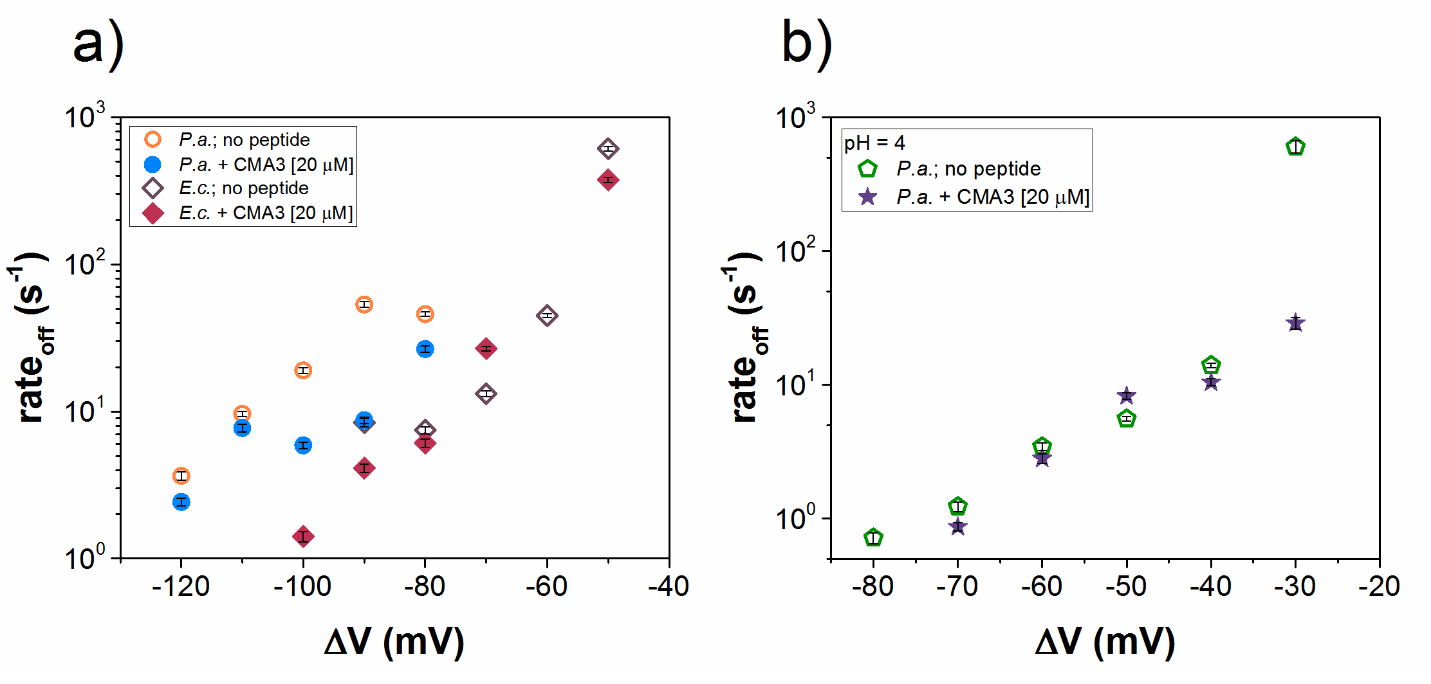


**Figure S4** Dissociation kinetics of *Pseudomonas aeruginosa* (*P.a*) and *Escherichia coli* (*E.c.*) from α-HL, as a function of the applied voltage. (a) Voltage dependence of the dissociation rate rate_off_ (rate_off_ = τ_off_^-1^) of bacterial cells from the α-HL nanopore for *P.a.* and *E.c* in the absence (open circles and diamonds, respectively) and in the presence of a synthetic antimicrobial peptide (CMA3) added on the *trans* chamber at a bulk concentration of 20 µM (closed circles and diamonds, respectively), at pH = 7. Panel (b) shows the voltage dependence of rate_off_ in the absence (open pentagons) and in the presence of 20 µM CMA3 added on the *trans* chamber (closed stars), at pH = 4.


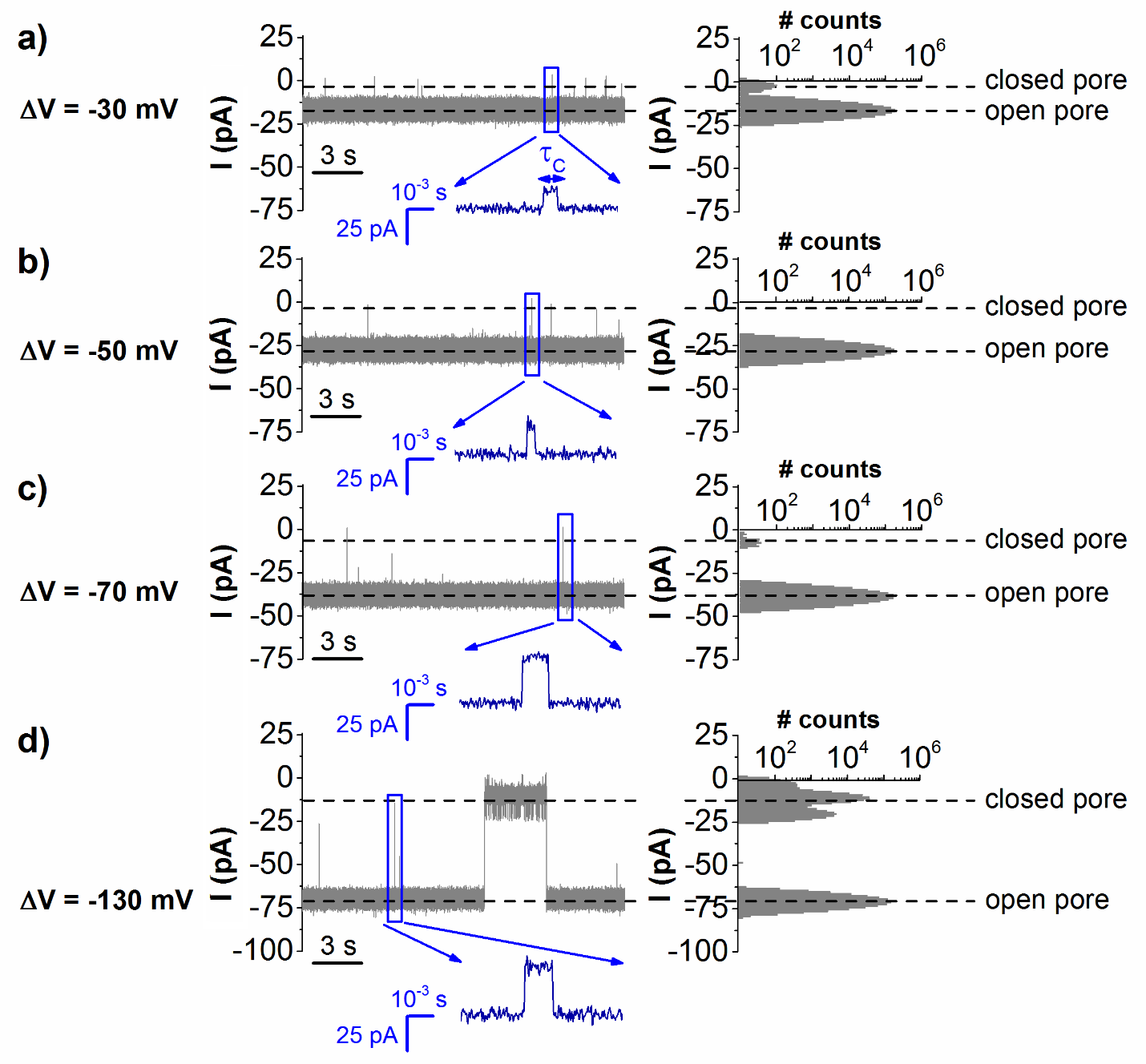


**Figure S5.** Original current recordings through the α-HL protein pore in the absence of added peptide and bacteria, at ΔV = -30 mV (a), -50 mV (b), -70 mV (c), and -130 mV (d), respectively, demonstrating the occurrence of stochastic changes in the ion current through the protein at low pH. τ_C_ in the inset of panel a denotes a representative time interval spent by the protein in such the closed state, illustrated in each of the left panels. The corresponding amplitude histograms shown in the right panels indicate the value of the ion current through the open and the closed α-HL pore, respectively. Recordings were performed at pH = 4.
